# Implication of the Type IV Secretion System in the Pathogenicity of *Vibrio tapetis*, the Etiological Agent of Brown Ring Disease Affecting the Manila Clam *Ruditapes philippinarum*

**DOI:** 10.3389/fcimb.2021.634427

**Published:** 2021-04-29

**Authors:** Alexandra Rahmani, François Delavat, Christophe Lambert, Nelly Le Goic, Eric Dabas, Christine Paillard, Vianney Pichereau

**Affiliations:** ^1^ Univ Brest, CNRS, IRD, Ifremer, UMR 6539 LEMAR, Plouzane, France; ^2^ UMR CNRS 6286 UFIP, University of Nantes, Nantes, France

**Keywords:** Brown Ring Disease, *Vibrio tapetis*, *Ruditapes philippinarum*, hemocytes, type four secretion system, rounding phenotype, colonization, mutagenesis

## Abstract

*Vibrio tapetis* is a Gram-negative bacterium that causes infections of mollusk bivalves and fish. The Brown Ring Disease (BRD) is an infection caused by *V. tapetis* that primarily affects the Manila clam *Ruditapes philippinarum*. Recent studies have shown that a type IV secretion system (T4SS) gene cluster is exclusively found in strains of *V. tapetis* pathogenic to clams. However, whether the T4SS is implicated or not during the infection process remains unknown. The aim of this study was to create and characterize a *V. tapetis* T4SS null mutant, obtained by a near-complete deletion of the *virB4* gene, in order to determine the role of T4SS in the development of BRD. This study demonstrated that the T4SS is neither responsible for the loss of hemocyte adhesion capacities, nor for the decrease of the lysosomal activity during BRD. Nevertheless, we observed a 50% decrease of the BRD prevalence and a decrease of mortality dynamics with the Δ*virB4* mutant. This work demonstrates that the T4SS of *V. tapetis* plays an important role in the development of BRD in the Manila clam.

## Introduction

Mass mortalities have occurred in the last decades impacting bivalves in hatcheries as well as in natural beds. Many *Vibrio* species are involved in these plagues and are known to be important pathogens in aquaculture (Travers et al., 2015). *Vibrio tapetis* (Borrego et al., 1996) is the etiological agent responsible for the Brown Ring Disease (BRD) (Paillard and Maes, 1990; Paillard et al., 1994) primarily affecting the Manila clam *Ruditapes philippinarum* (Linnaeus, 1758). BRD is characterized by a brown organic deposit, in the inner face of the shell, between the pallial line and the edge of the shell, which can often be observed in Manila clams during the winter and spring periods but is present all year long (Paillard et al., 2014; Paillard, 2016), mostly on the European Northern coast. BRD is a cold water disease based on the fact that *V. tapetis* is virulent only at low temperatures, with an optimum infection temperature of 14°C (Paillard et al., 1994; Paillard, 2004a).

The effects of BRD infection on *R. philippinarum* have been well documented. The pathogen enters the pallial cavity of clams and then colonizes and degrades their periostracum to penetrate into extrapallial fluids (EPFs) where they can spread (Paillard et al., 1994; Paillard, 2004a). Hemocytes, which are the clam’s immune system cells, are recruited to the site of infection within the extrapallial fluids to eliminate the pathogen (Allam et al., 2000). During a typical infection, hemocytes phagocytize foreign bacteria and mobilize the lysosome to eliminate them (Al-Khalaifah and Al-Nasser, 2018). These immune cells are characterized by pseudopods, which are actin-rich membrane expansions, that make them able to catch and internalize bacteria. During the development of BRD, *V. tapetis* cells are phagocytized by hemocytes, but once internalized, they inhibit several cellular functions such as actin polymerization or phagosome–lysosome fusion, thereby avoiding degradation (Choquet et al., 2003; Paillard, 2004a; Al-Khalaifah and Al-Nasser, 2018; Rahmani et al., 2019). Moreover, Reactive Oxygen Species (ROS) production is reduced in infected hemocytes (Paillard, 2017). These bacteria-mediated inhibitions of critical cellular functions lead to the multiplication of *V. tapetis* within the hemocytes, which are ultimately lysed when bacteria reach high densities, allowing *V. tapetis* cells to spread in EPFs.

However, while knowledge is improving regarding the behavior of clams and clam hemocytes upon infection by *V. tapetis*, very little is known about the precise mechanisms linked to the virulence of the pathogen. A characterization of the *V. tapetis’* secretome upon exposure to Manila clam hemocytes allowed the identification of several proteins potentially involved in pathogenicity (Madec et al., 2014). *V. tapetis* is also able to produce several virulence factors potentially involved in its pathogenic capacity such as hemolysins, cytotoxins and exotoxins (Allam and Ford, 2006). It has also been recently demonstrated that the structures of the biofilms formed by different *V. tapetis* strains vary, depending on whether they are clam- or fish- pathogens (Rodrigues et al., 2015).

The genomes of various strains of *V. tapetis* have recently been sequenced and compared. Interestingly, this analysis showed that the Type IV Secretion System (T4SS) cluster is exclusively found in strains of *V. tapetis* reproducing BRD in clams and mainly isolated from infected clams, and not in the ones isolated from fish (Dias et al., 2018). Furthermore, a diagnostic method has recently been developed to quantify *V. tapetis* in EPFs relying on the presence of the *virB4* gene (Bidault et al., 2015). The T4SS is a delivery system, which can transfer proteins, but also DNA, directly into recipient cells (which can be either prokaryotes or eukaryotes) (Christie and Cascales, 2005). T4SSs were first described in the plant pathogen *Agrobacterium tumefaciens* (Stachel and Nester, 1986) and are well known to be involved in pathogenicity in multiple infections such as Brucellosis (*Brucella* spp.*)* or Legionnaires’ disease (*Legionella pneumophila*) (Voth et al., 2012). One of the characteristics of T4SSs is the presence of a conserved ATPase, called VirB4 in *A. tumefaciens*. VirB4 is one of the two ATPase of this T4SS, being essential for both T4SS structure and activity (Kerr and Christie, 2010; Waksman, 2019).

Based on the presence of the T4SS cluster only in *V. tapetis* strains infecting clams together with the demonstrated role of T4SS in the pathogenicity of many bacteria, this secretion system is therefore an attractive candidate to be tested to understand the pathogenicity mechanisms used by *V. tapetis* during infection of the Manila clam. Furthermore, a recent RNAseq experiment demonstrated the expression of 8 *virB* genes belonging to *V. tapetis* CECT4600 T4SS, highlighting that this is an active cluster (Rodrigues et al., 2018). The best way to test such a hypothesis is to delete the corresponding genes. However, despite laborious efforts, only one deletion mutant (*δdjlA*) of *V. tapetis* CECT4600 infecting clams has been generated so far (Lakhal et al., 2008) and has shown its role on hemocyte cytotoxicity. This *δdjlA* mutant was not able to induce mortality or BRD development in clams, but this result may be a consequence of the growth defect phenotype of the mutant (Lakhal et al., 2008).

**Figure 1:**
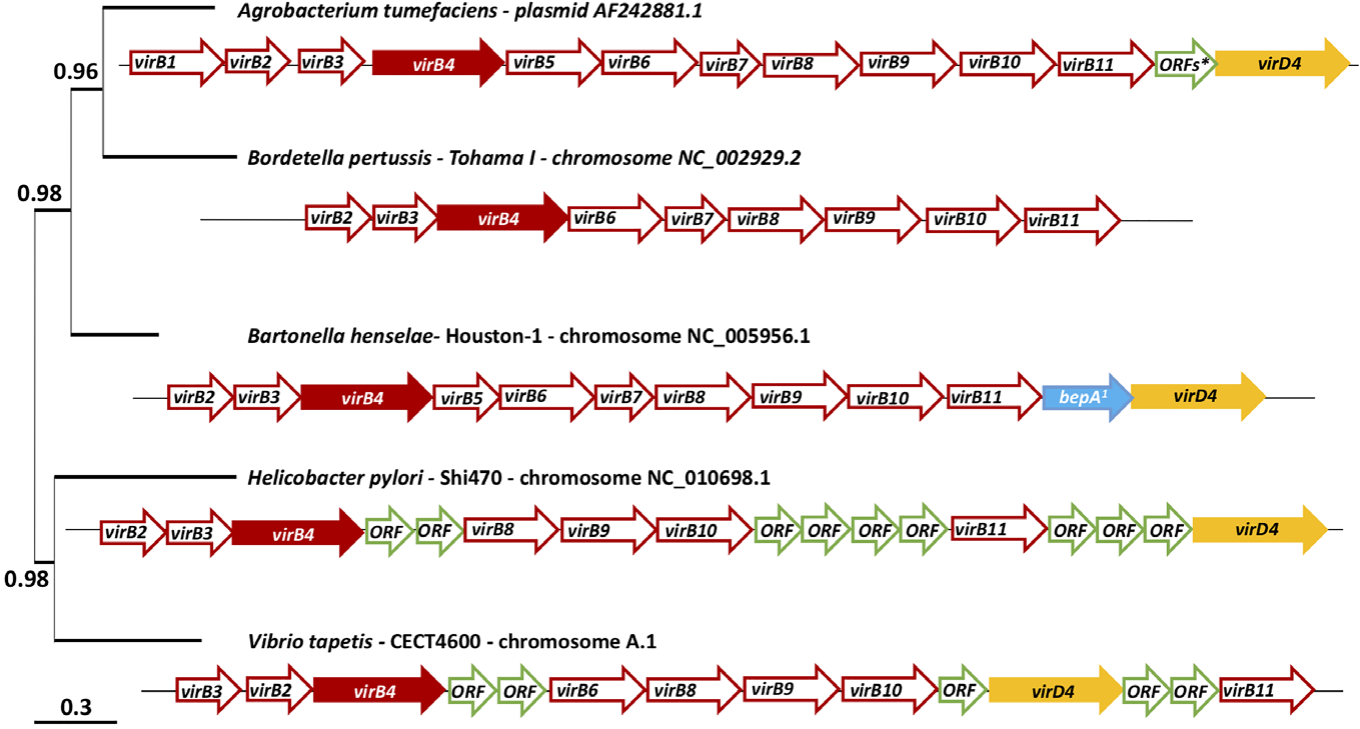
virB/D4 and homologues genes cluster coding the T4SS of *Agrobacterium tumefaciens*, *Bordetella pertussis*, *Bartonella henselae*, *Helicobacter pylori* and *Vibrio tapetis* and phylogenetic tree of these five strains. The phylogenetic tree was constructed using the gene virB4 and the software Phylogeny, “One click” mode (Dereeper et al., 2008). *This arrow represents 15 ORFs. a: bepA: bartonella effector protein. A detailed table (**Table S1**) is available on **Supplementary Material** with the name of the sequences available on MaGe: MicroScope plateform (Vallenet et al., 2009) and used to represent these cluster.

**Figure 2:**
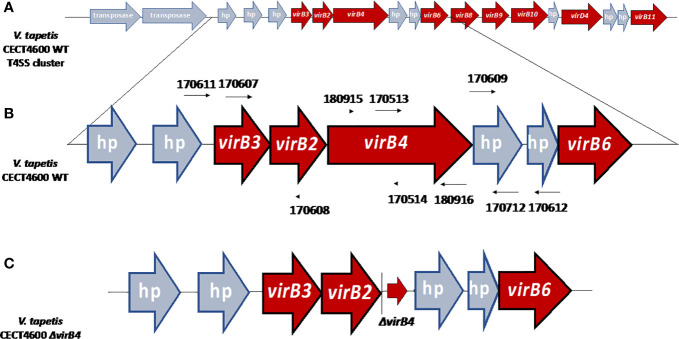
**(A)** Organization of the T4SS gene cluster of *V. tapetis* CECT4600 located in the chromosome A (accession number NZ_LT960611.1). **(B)** Genetic environment before the deletion of the *virB4* gene with the positions of primers used in this study. UP and DOWN fragments have been amplified using primers 170,607–170,608 and 170,609–170,712 respectively. **(C)** Genetic environment after the deletion of *virB4* gene, the Δ*virB4* arrow corresponding the three first and the three last codons of virB4 gene, allowing the formation of a non-functional peptide of six amino-acids, limiting the risk of polar effects. hp, hypothetical protein. Based on the description of *V. tapetis* CECT4600 T4SS in Dias et al., 2018.

In this study, we aimed at clarifying the role of the T4SS in the development of the BRD, by deleting the *virB4* gene of *V. tapetis* CECT4600. This gene is surrounded by *virB2* (upstream) and *virB6* (downstream, preceded by two hypothetical proteins) genes (**Figures 1** and **2**) (Dias et al., 2018). A targeted mutagenesis approach has been developed, which led to the deletion of the *virB4* gene. We could demonstrate that the T4SS, while not being essential to induce vibriosis, plays an important role in BRD development in the Manila clam. This approach and the generation of deletion mutants will help to better understand the genes, and therefore the mechanisms, used by the pathogen during the development of BRD.

## Materials and Methods

### Bacterial Strains, Plasmids and Culture Conditions

Bacterial strains used in this study are described in **Table 1**. Plasmids and primers used in this study are listed in **Tables 2** and **S2**, respectively. *Escherichia coli* strains were routinely grown at 37°C in LB medium. *V. tapetis* CECT4600 and *ΔvirB4* were grown in LB containing 20 g/l (final concentration) of NaCl (LBS) or in Zobell medium (Zobell, 1941) at 18°C ± 2°C. We used LBS for molecular biology because *V. tapetis* grows better in this rich medium and is therefore better suited for mutagenesis; Zobell was used for *in vitro/in vivo* experiments, because it allows a good reproducibility, allowing the present results to be compared with previous studies on this strain. When necessary, the following components were added: Kanamycin (Km, 100 µg/ml), Chloramphenicol (Cm, 5 µg/ml for *E. coli* (Cm^5^); 1 (Cm^1^) or 5 µg/ml (Cm^5^) for *V. tapetis*), Trimethoprim (Trim, 10 µg/ml), Diaminopimelic acid (DAP, 0.3 mM), D-Glucose (D-Glc 1%), L-Arabinose (L-Ara 0.2%) or agar (15 g/l). Growth parameters of strains CECT4600 and *ΔvirB4* were determined by using a microplate reader BioscreenC (Bioscreen, Turku, Finland) in both LBS and Zobell media at 18°C with agitation, in 100-well microplates, after 24 h of growth.

**Table 1:** Bacterial strains used in this study.

Strains	Strains collection number	Description	Reference
***E. coli* strains**			
DH5α λpir	9	*supE44, ΔlacU169 (ΦlacZΔM15), recA1, endA1, hsdR17, thi-1, gyrA96, relA1, λpir phage lysogen*	Lab strain
GEB883	62	*E. coli K12 ΔdapA::erm, pir, RP4-2, ΔrecA, gyrA462, zei298::Tn10, Ery^R^, Tet^R^, DAP-, conjugative strain*	(Nguyen et al., 2018)
*E. coli* GEB883 + pFD055	88	*E. coli* GEB883 containing pFD055	This study
*E. coli* GEB883 + pEVS104	102	*E. coli* GEB883 containing pEVS104	This study
			
*E. coli* DH5α λpir + pAR003	234	*E. coli* DH5α λpir containing pAR003	This study
			
***V. tapetis* strains**			
CECT4600	2	*V. tapetis* reference strain (wild type) isolated form *R. philippinarum*	(Borrego et al., 1996)
*ΔvirB4*	177	CECT4600 strain in which the *virB4* gene has been deleted	This study
*ΔvirB4+ pAR003*	268	Strain 177 containing pAR003	This study

**Table 2:** Plasmids used in this study.

Plasmids	Characteristics	Reference
pGEM-T® Promega	*Amp^R^, lacZ*	Promega
pLP12	*oriT* RP4*, oriVR6K, P_BAD_-vmi480, CmR*, suicid plasmid in *V. tapetis*, replicative plasmid in *E. coli* λpir strains	Luo et al. (2015)
pFD055	pLP12 plasmid containing the upstream and downstream regions of the *virB4* gen*e* of V. tapetis CECT4600	This study
pJLS199	*oriT* RP4*, oriV R6K*, Trim^R^, Replicative in *V. tapetis* and in *E. coli*. Derived from (Stoudenmire et al., 2018)	Delavat et al. (2018)
pEVS104	*Km^R^*, replicative plasmid in *E. coli*, helper plasmid for conjugation	Stabb and Ruby (2002)
pVSV102	*Km^R^*, containing a *gfp* gene, Replicative in *V. tapetis* and in *E. coli*	Dunn et al. (2006)
		
pFD085	Derivative of pJLS199, in which the *mcherry* gene has been removed (using *Eag*I), and in which the RBS region of the *gfp* gene has been replace by the one found upstream of the *gfp* gene of pVSV102. Replicative plasmid in *E. coli* and *V. tapetis*, Trim^R^	This study
pFD086	Derivative of pFD085 containing the P_lac_ promotor of pVSV102 upstream of the *gfp* gene (*XhoI/Xba*I), replicative plasmid in *E. coli* and *V. tapetis*, Trim^R^	This study
pAR003	Derivative of pFD086 in which the *gfp* gene has been replaced by the *virB4* gene (*Xba*I/*Bam*HI), expressed from the P*_lac_* promoter. Replicative plasmid in *E. coli* and *V. tapetis*. Trim^R^	This study

All in silico plasmid maps and sequences are available upon request.

### Construction of a *ΔvirB4* Deletion Mutant

The *virB4* gene was deleted by double homologous recombination using a pLP12-derived suicide plasmid (Luo et al., 2015), under specific culture and mating conditions. Briefly, two fragments of 820 and 799 bp located upstream (UP) and downstream (DOWN) of the *virB4* gene of *V. tapetis* CECT4600, respectively, were PCR-amplified using primers 170,607–170,608 and 170,609–170,712, respectively. The two DNA fragments were fused by Splicing by Overlap Extension (SOEing) PCR using primers 170607 and 170612, giving a single 1.6-kbp fragment UP-DOWN (UD-fragment) that was inserted into the pGEM-T plasmid (Promega, Madison, WI, USA) by ligation with T4 DNA ligase (Promega) according to the manufacturer recommendations. The integrity of the UD-fragment was verified by sequencing (Eurofins Genomics company, Ebersberg, Germany). And then released from pGEM-T using *Eco*RI and *Nhe*I (Promega, Madison) and ligated into the pLP12 plasmid digested with the same enzymes. The ligation mixture was transformed by heat shock into competent cells of *E. coli* DH5α λpir, and a clone containing the pLP12 plasmid in which the UD-fragment had been inserted (named pFD055) was stored at −80°C. The purified pFD055 plasmid was then transferred to *E. coli* GEB883 by heat shock transformation (strain 88). *E. coli* GEB883 was also transformed with the pEVS104 plasmid in order to obtain the helper strain (strain 102, **Table 1**) used in this study.

The pFD055 plasmid was transferred to *V. tapetis* CECT4600 by a triparental mating procedure. Briefly, strains 88, 102 and 2 (**Table 1**) were mixed at a 1:1:5 ratio. Each strain was harvested one after another, centrifuged (7,168×*g*, 3 min) and the supernatant was removed in order to avoid exposure to antibiotics for the next strain added. The cell pellet was finally resuspended in 20 µl of LBS + D-Glc + DAP and spotted on a cellulose acetate filter (0.22 µm, Sartorius Stedim biotech, Goettingen, Germany) deposited on a LBS + D-Glc + DAP plate. This plate was then incubated at 25°C for 48 h. The spot was then resuspended with 1 ml LBS, and 100 µl were plated on both LBS + D-Glc + Cm^5^ and LBS + D-Glc + Cm^1^. The remaining suspension was centrifuged (7,168x*g*, 3 min), resuspended in 200 µl of LBS, split into two 100 µl fractions which were plated on the same culture media. All plates were incubated at 20°C for 5 days.

The obtained colonies were re-streaked on LBS + D-Glc + Cm^1^ and the site of chromosomal insertion of pFD055 was determined by PCR, using 170,611–170,902 (for a recombination between UP fragments) and 170,901–170,612 (for a recombination between DOWN fragments).

After confirmation of plasmid integration, the single clone that had integrated pFD055 was cultivated overnight in LBS. The culture was serially diluted, and 100 µl were plated on LBS + L-Ara to induce expression of the Vmi480 toxin encoding gene, located in pLP12 (Luo et al., 2015), to select for excision of pFD055 by a second recombination. Following incubation at 20°C, the obtained colonies were tested for deletion of the *virB4* gene by using primers 170,611–170,612 [expected size 1,759 bp in the mutant, 4,285 bp in the wild type (WT)] and 170,513–170,514 (no PCR product in the mutant, 173 bp in the WT). A *V. tapetis* mutant strain deleted for the *virB4* gene was stored at −80°C (strain 177).

### Complementation of the *ΔvirB4* Deletion Mutant

The full coding sequence of the *virB4* gene of *V. tapetis* CECT4600 (from the ATG start codon to the TGA stop codon) was PCR-amplified using 180,915–180,916, cloned into the pGEM-T vector and verified by sequencing. The obtained construction, as well as the pFD086 plasmid, were then both digested using *Xba*I and *Bam*HI, the appropriate DNA fragments were purified from the electrophoresis gel (NucleoSpin Gel and PCR Clean-up kit, Macherey-Nagel, Hoerdt, France) and ligated. The replacement of the *gfp gene* in pFD086 by *virB4* was checked by using primers 180,401–170,514 (expected size 2,848 bp). The obtained pAR003 plasmid was then transformed into *V. tapetis* Δ*virB4* (strain 177) by triparental mating as described earlier, by mixing the strains 234, 102, and 177 (**Table 1**) at 18°C, for 24 h. The mating mixture was plated on LBS + Trim and the presence of pAR003 in *V. tapetis* Δ*virB4* was confirmed by using 180,401–170,514 (expected size 2,848 bp) to obtain the strain 268.

### *In Vitro* Virulence Assays on Manila Clams

The *in vitro* virulence assay was performed, following a standardized protocol developed in order to test the ability of *V. tapetis* strains to induce a rounding of hemocytes of the Manila clam (Choquet et al., 2003). Briefly, Manila clams from the SATMAR shellfish aquaculture site in Marennes (Charente-Maritime, France) were acclimatized in oxygenated seawater at 14°C. Hemolymph was harvested directly from the adductor muscle of each individual, and the quality of hemocytes was checked by microscopical observation (presence of pseudopods, number of rounded hemocytes). Good quality hemolymph samples were pooled and the hemocytes enumerated by using a Malassez cell. Hemolymph was exposed to a bacterial suspension of *V. tapetis* CECT4600 or its derivatives (**Table 1**, plated on Zobell agar) in at least three replicates and two independent experiments. 100 μl of hemolymph were added in 24-well plates, followed by 100 μl of bacterial suspension [in Filter-Sterilized Seawater (FSSW)], to obtain a 25/1:bacteria/hemocyte ratio);. For the controls, the bacterial suspension was replaced by 100 μl of FSSW. The flow cytometry analysis was coupled with the LysoTracker assay described below, and results were expressed as the ratio of non-adherent hemocytes in exposed vs control samples.

### LysoTracker Assays on Manila Clams Hemocytes

The LysoTracker probes (Invitrogen™, Paisley, UK) are fluorescent acidotropic probes for labeling and tracking acidic organelles in live cells, according to the manufacturer’s definition. We used this method to characterize the amounts of acidic organelles in hemocytes exposed to the different strains of *V. tapetis*, or FSSW for control. This LysoTracker assay, performed during the *in vitro* virulence test, has recently been developed in order to correlate the rounding phenotype to the cell content in acidic organelles by performing both tests (i.e. virulence test and LysoTracker assay) on the same samples (Rahmani et al., 2019). This last study also highlight the highly probable link between the amount of acidic organelles and the fusion between the phagosome and lysosome using cytometry experiment with the LysoTracker coupled with a transcriptomic analysis in the case of BRD (Rahmani et al., 2019).

The virulence test was performed as described above. After 1 h of incubation at 18°C, 4 µl of 50 µM LysoTracker Red DND-99 (Invitrogen™, emission from 550 to 700 nm, final concentration 1 µM) was added to each well. After 2 more hours of incubation at 18°C, the contents of the wells were then transferred to 5-ml cytometry polystyrene Falcon tubes (BD Biosciences, San Jose, CA, USA).

Flow cytometry analyses were performed by using a BD FACSVerse flow cytometer using its blue laser (488 nm) as an excitation source. The mean red fluorescence level (LysoTracker fluorescence linked to acidic organelles) of the selected hemocytes was measured using the PerCP-Cy5.5 detector of the flow cytometer (700/54 nm).

Subsequently, 2 µl of a 100× dilution in ultra-pure water of a commercial solution of SYBR–Green I nucleic acid gel stain 10,000× in DMSO (Molecular Probes by Life Technology, CA, USA) were added in each tube for 10 min at room temperature, in dark conditions, before measurement of the green fluorescence. Addition of SYBR-Green allows the selection of hemocytes by FITC detector of the flow cytometer (527/32 nm) and then the quantification of the number of non-adherent hemocytes in our samples.

This second flow cytometry analysis allowed measurement of both the green and red fluorescences, and was performed by using excitation wavelengths of 488 and 640 nm, respectively. Results were expressed in mean red fluorescence level per hemocyte (arbitrary units, AU) measured with the APC detector of the flow cytometer (660/10 nm) instead of the PerCP-Cy5.5 detector (700/54 nm) to overcome a possible SYBR-Green fluorescence overlap.

To determine whether the differences between the ratio of non-adherent hemocytes and the amount of acidic organelles in exposed and controls samples are statistically significant, a Wilcoxon test was performed.

### *In Vivo* Virulence Assays on Manila Clams

For the *in vivo* experiments, two modes of infection were used: (i) inoculation of a fixed number of bacteria into the pallial cavity, which results in BRD symptoms after four weeks (Oubella et al., 1994) and (ii) injection of *V. tapetis* strains into the adductor muscle, which induces clam mortalities that start 3 to 5 days after injection (Allam et al., 2002).

- The day before injection, clams were removed from water to allow them to open enough for injection the next day. 150 clams were injected with 100 µl of a suspension (5 x 10^7^ CFU/animal) of strains *V. tapetis* CECT 4600 or Δ*virB4*, or with 100 µl FSSW as the control. All injected clams were left for 6 h out of water to promote the colonization of the periostracal lamina by *V. tapetis*. Animals were then placed into tanks (50 animals per replicate, in triplicate) equipped with a bubbler, at 14°C. Animals were not fed during the experiment and water was changed once after 2 weeks post injection (p.i.). The replicates were injected on 3 independent days. After 4 weeks p.i., animals were sacrificed and shells were kept and analyzed according to the method developed by Paillard and Maes (1994) to determine the BRD prevalence in each condition. To determine whether the BRD prevalence was statistically significant between these conditions, a Chi-2 test with 2 degrees of freedom was performed.
- 30 animals (ten clams per replicate) were injected in the adductor muscle with either a bacterial suspension (5 x 10^7^ CFU/animal) of strains *V. tapetis* CECT4600, *V. tapetis* Δ*virB4* or *V. tapetis* Δ*virB4* +pAR003, respectively for the experimental conditions, or with 100 µl FSSW as a control. Animals were then placed into tanks equipped with a bubbler, at 14°C. Dead animals were enumerated and collected throughout the experiment, thus enabling determination of the mortality rates. Data represented survival rates for each condition. Statistical analyses were performed using Kaplan–Meier survival analysis and a Wilcoxon test on R software.

### Bioinformatics and Biostatistics

Handling of DNA sequences was performed by using the SnapGene software version 5.1.7. (Insightful Science). Phylogenetic analysis was also performed, based on the *virB4* genes of 4 bacterial pathogens that carry the same type of T4SS than *V. tapetis* CECT4600*: Agrobacterium tumefaciens, Bartonella henselae, Helicobacter pylori* and *Bordetella pertussis*. This analysis was conducted using the software Phylogeny.fr (Dereeper et al., 2008) that used for alignment: *MUSCLE*, for curation: *Gblocks*, for phylogeny: *PhyML* and for graphic representation: *TreeDyn*. The Atlas T4SS tools available at www.t4ss.lncc.br/ (Souza et al., 2012) was also used to highlight the annotation corresponding to the sequence VTAP_v1_a3555, that might correspond to the *virB5* gene of *V. tapetis* CECT4600 T4SS cluster. All biostatistics analyses were performed using the R software (Core Team, R., 2017).

## Results

### Characterization of T4SS Genes Cluster of 5 Bacterial Pathogens and Phylogeny of the Gene *virB4* Between These Strains

**Figure 1** schematizes the T4SS gene cluster of *V. tapetis* CECT4600 as compared to four other pathogenic bacterial species, including the pathogen *A. tumefaciens* (metadata are also available in **Table S1**). The phylogenetic tree in **Figure 1** also allows the representation of phylogenetic distance between the gene *virB4* of each of these strains. *V. tapetis* CECT4600’s T4SS is composed of genes *virB2, 3, 4, 6, 8, 9, 10, 11* and *virD4* (**Figures 1** and **2**) (Dias et al., 2018). In the strain CECT4600 genome, genes *virB1* and *viB7* are missing as compared to the T4SS of *A. tumefaciens*. According to our previously published annotation (Dias et al., 2018), the *virB5* gene is also missing. As we suspect that the sequence VTAP_v1_a3555 might correspond to the *virB5* gene, we further analyzed this sequence and revealed that for this sequence, which is located between *virB4* and *virB6* genes, the ORF size, position, sequence variability and predicted N-terminal cleavage peptide are good indications that probably this is the *virB5* gene. However, we found very low homology between the suspected VirB5 protein sequence and other VirB5 characterized proteins of (the highest homologies being obtained with that of *Campylobacter jejuni*, i.e. Blast E.value: 0.003, and 28% identity and 49% homology over a 99 aminoacids region) which might also indicate that further physiological analysis should be performed in order to confirm this annotation.

### Deletion of the *virB4* Gene in *V. tapetis* CECT4600 and Physiological Characterization of the *ΔvirB4* Mutant

In order to determine the role of the T4SS in the virulence of *V. tapetis* CECT4600, the T4SS-conserved gene *virB4* was chosen as a target for gene deletion. *V. tapetis* death occurs at around 30°C and its optimal growth temperature is 18°C, which is low compared to that of *E. coli* (Borrego et al., 1996). Based on the recent observation that the growth of *Vibrio* strains under supra-optimal conditions increases the chance of transferring foreign DNA into marine bacteria (Delavat et al., 2018), matings between *V. tapetis* CECT4600 and *E. coli* carrying the suicide plasmid pFD055 were performed at 25°C. After selection of chloramphenicol-resistant clones, one colony was obtained, that had integrated the pFD055 plasmid into its genome. This clone was tested by PCR which showed an integration of pFD055 by recombination within the “DOWN” region. After selection for the excision of the pFD055 plasmid by induction of the Vmi480 toxin with L-Arabinose, some of the obtained colonies were tested by colony-PCR to determine the genotype of the clones, regarding the presence of *virB4*. Ultimately, clones in which the *virB4* gene was deleted in *V. tapetis* CECT4600 were obtained and one clone (**Figure S1**) was stored at −80°C for subsequent analyses.

In order to characterize the effect of the *virB4* deletion on *V. tapetis* CECT4600, the doubling time of both strains was compared, under both LBS and Zobell culture conditions in a microplate. The growth rates of both strains in LBS were around two times slower than in Zobell, even though the final cell densities were higher when grown in LBS (**Table S3**). Statistical analyses of doubling time and lag phase were performed by Wilcoxon tests. The doubling time of the *virB4* mutant as compared to the WT, was higher in LBS (*p = 0.016*) but not in Zobell (*p = 0.06*) and the lag phase was longer in LBS medium as compared to growth in the Zobell medium (*p = 0.0002)* for both strains. Therefore, although the absence of the *virB4* gene leads to a slight increase in the doubling time of *V. tapetis* in LBS, this deletion does not significantly modify the growth of the mutant in Zobell medium.

### Loss of Hemocytes’ Adhesion Properties, Observed During *V. tapetis* Infection, Is T4SS Independent

**Figure 3:**
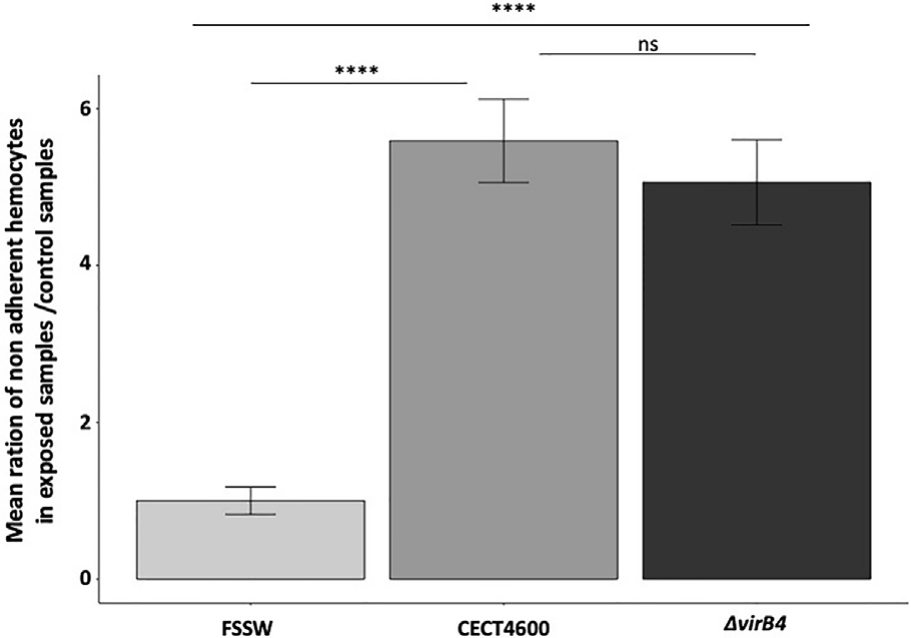
Effect of the *ΔvirB4* mutation on the adhesion properties of clam hemocytes. Results are presented by mean ratio of non-adherent hemocytes (i.e. round hemocytes) on exposed samples/control samples. ****p value <10^−4^ (student test), ns, not significative. Cytograms and fluorescence pics are available in **Figure S2**. Flow repository ID: FR-FCM-Z3WT.

In order to characterize the importance of the T4SS in the virulence of *V. tapetis* CECT4600 toward *R. philippinarum* hemocytes, an *in vitro* adherence test of clam hemocytes was performed, comparing the WT- and the Δ*virB4*- strains both in LBS (data not shown) and in Zobell media (**Figures 3** and **S2**)*. E*arlier works showed that *V. tapetis* CECT4600 induces a loss of hemocyte adhesion properties, by causing rounding of the cells and de-structuration of the actin cytoskeleton (Paillard, 2004b; Rahmani et al., 2019). Our results showed that the ratio of non-adherent hemocytes in exposed versus control samples was increased by 5.6 fold when hemocytes were exposed to *V. tapetis* CECT4600, as compared to hemocytes exposed to FSSW (**Figures 3** and **S2**). Interestingly, inactivation of the T4SS did not lead to a decrease in this ratio (**Figures 3** and **S2**) neither in Zobell or LBS. These results strongly suggest that the T4SS of *V. tapetis* does not contribute to the pathogen-induced loss of hemocyte adhesion properties.

### The T4SS Is Not Solely Responsible for the Inhibition of the Lysosomal Activity in *V. tapetis* Infected Hemocytes

**Figure 4:**
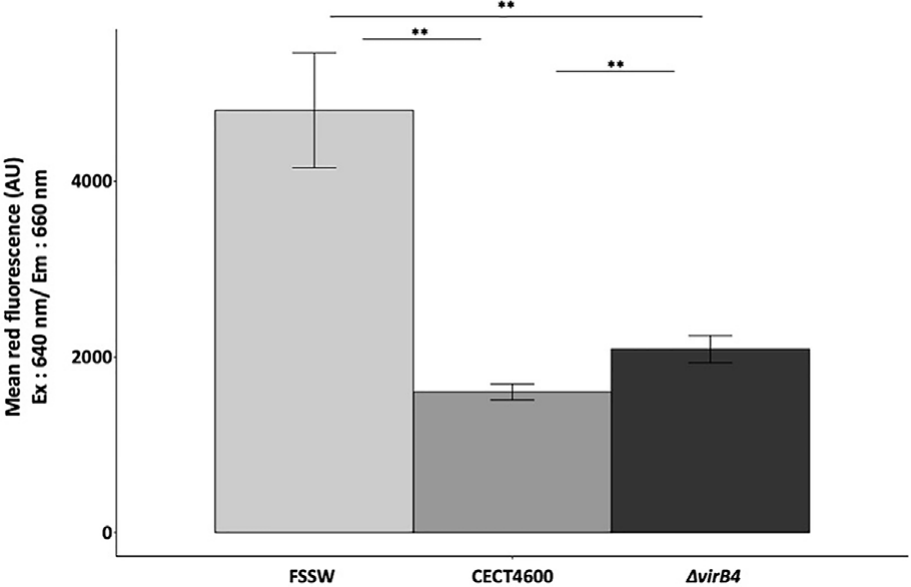
Measurement of acidic organelles amount by flow cytometry using LysoTracker Red DND-99^®^. Excitation of the probe has been done at 640 nm, as specified in the x axis. Mean fluorescence measured in this range of excitation laser in y axis for each strain tested. **p.value <0.004 (Wilcoxon test). Cytograms and fluorescence pics are available in **Figure S2**. Flow repository ID: FR-FCM-Z3WT.

To test whether the T4SS is involved in the inhibition of the phagosome–lysosome fusion, we characterized the amounts of acidic organelles in hemocytes exposed to *V. tapetis* or the *virB4* mutant in comparison with exposure to FSSW, by using the LysoTracker Red DND-99 in flow cytometry. Hemocytes exposed to *V. tapetis ΔvirB4* show significantly more acidic organelles as compared to CECT4600 (*p = 0.004*), highlighting a role of the T4SS in the decrease of lysosomal activity (**Figures 4** and **S2**). However, the T4SS is not solely responsible, since the *virB4* mutant still shows a 2-fold decrease in the amount of acidic organelles, as compared to the control hemocytes (*p = 0.003*).

### The T4SS of *V. tapetis* Is Involved in BRD Development in the Manila Clam

**Figure 5:**
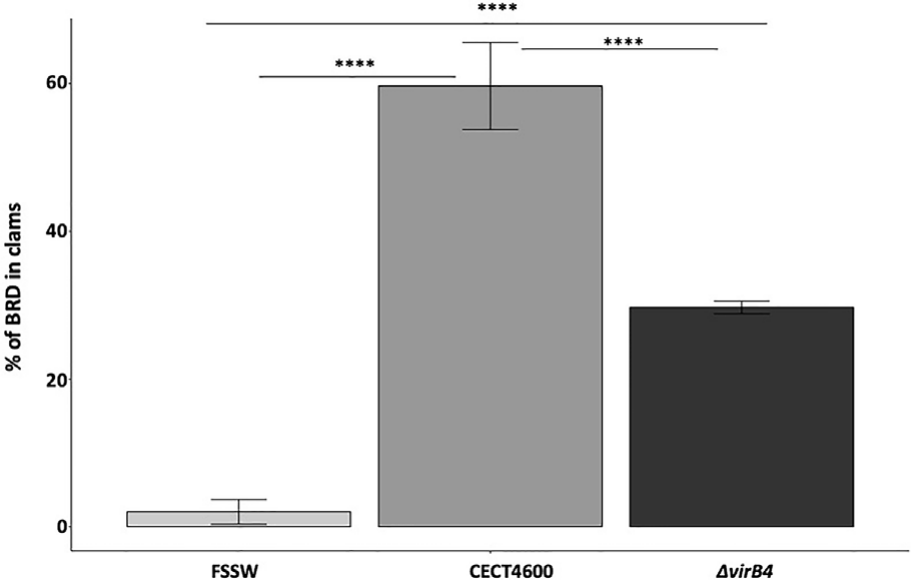
BRD prevalence after injection of V. tapetis strains CECT4600 and *ΔvirB4* in the pallial cavity of the Manila clam. ****p value <10^−4^ (Chi-2 test, 2df).

Our *in vitro* assays revealed that the T4SS does not seem to be involved in the cytotoxic effect (loss of hemocytes adhesion properties) and is not strictly required in the phagosome–lysosome inhibition of *V. tapetis* within hemocytes. To test whether the T4SS is important for BRD development in the Manila clam, injections of *V. tapetis* CECT4600 or *V. tapetis* Δ*virB4* into the pallial cavity of clams were performed. BRD prevalence was determined after 4 weeks post-injection (**Figures 5** and **S3**). The prevalence of BRD in control clams was very low (2%), confirming that clams were not infected before this experiment. After four weeks of infection, the prevalence of BRD observed in clams infected by *V. tapetis* CECT4600 was close to 60%, which is the expected prevalence already reported in several studies (Maes, 1992; Maes and Paillard, 1992; Paillard, 2004a; Paillard, 2004b). Interestingly, clams injected with a suspension of the Δ*virB4* mutant displayed a prevalence of BRD close to 30%, thus decreasing two-fold the prevalence of BRD in Manila clams. Consequently, our results show that the T4SS, although not essential, is a key contributor to BRD development.

### The T4SS of *V. tapetis* Is Involved in the Mortality Dynamics Observed After Injection of *V. tapetis* in the Adductor Muscle

**Figure 6:**
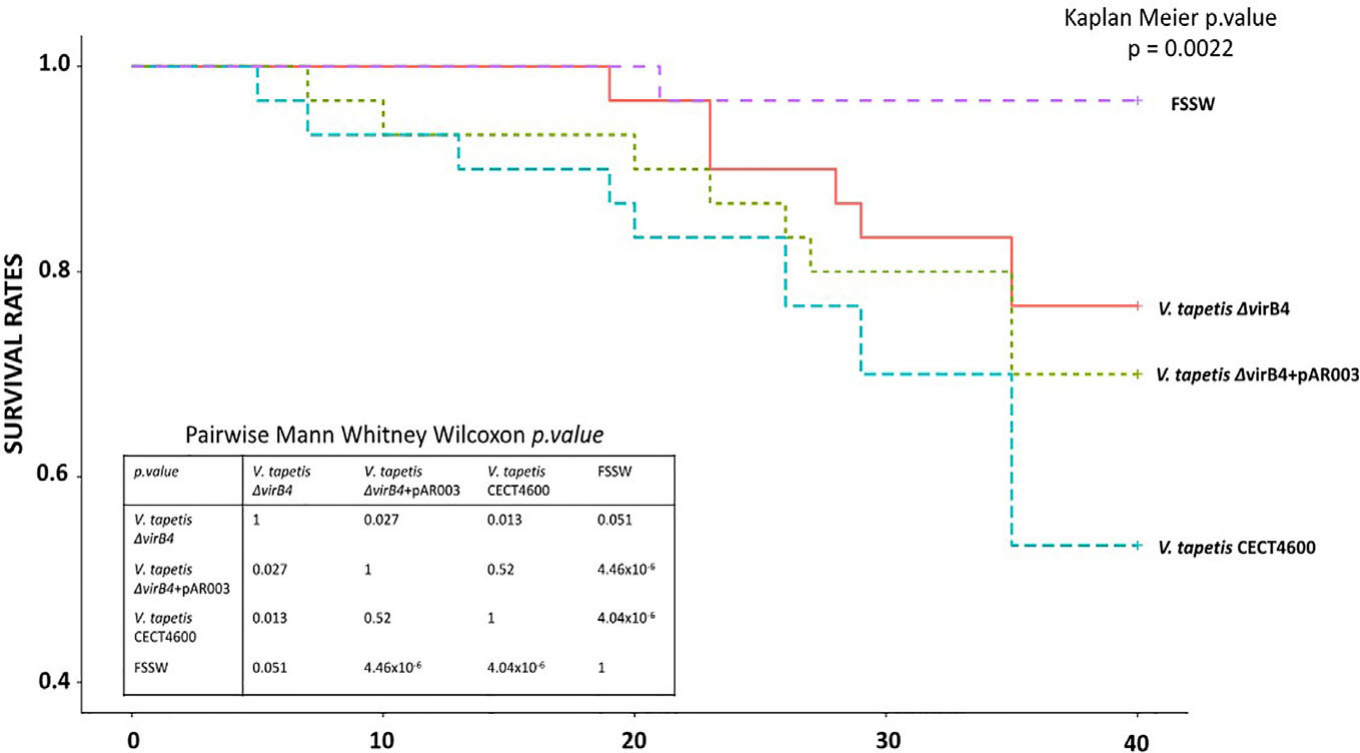
Mortality rates, using Kaplan–Meier analysis, observed after injection of strains *V. tapetis* strains CECT4600, *ΔvirB4* and *ΔvirB4*+pAR003 (30 clams/conditions) in the adductor muscle of the Manila clam *R. philippinarum*.

The T4SS is implicated in BRD formation, but it remained unknown whether this system is also important for the direct mortality of Manila clams. To answer this question, mortalities were monitored for 4 weeks after injection in the adductor muscle of Manila clams with *V. tapetis* strains. In this experiment, we expected mortality because the injection was performed in tissues (*i.e*. adductor muscle) and this protocol allows mortality within weeks as has been demonstrated previously (Allam et al., 2002; Choquet, 2004). Mortality rates observed in this experiment are presented in **Figure 6**. By using the Kaplan–Meier survival analysis, we showed that the mortality rates significantly differed among conditions (*p* = 0.002). Expectedly, the highest mortalities were observed when clams were injected with *V. tapetis* CECT4600. By contrast, *V. tapetis* Δ*virB4* displayed a lower virulence, as the mortality dynamics significantly differed from that of strains CECT4600 (*p* = 0.013). Specifically, the proportion of dead animals at the end of the experiment decreased from 43% to 23% following exposure to CECT4600 and Δ*virB4*, respectively. In order to confirm that this phenotype was effectively due to the Δ*virB4* deletion, we performed genetic complementation on *V. tapetis* Δ*virB4* by introducing into this mutant a plasmid carrying the *virB4* gene (from the ATG start codon to the TGA stop codon) cloned downstream of the Plac promotor and ribosome binding site (RBS) of the vector. The functional complementation of *virB4* in the Δ*virB4* mutant partially restored the virulence of this strain (**Figure 6**), with 30% of clam mortality after 5 weeks of infection, and a mortality dynamics that did not significantly differ from that observed for *V. tapetis* CECT4600 (*p* = 0.52). Thus, the functional complementation of *V. tapetis ΔvirB4* reproduced the mortality dynamics observed after injection in the adductor muscle of the *V. tapetis* CECT4600. This demonstrated that the attenuated virulence phenotype of *V. tapetis* Δ*virB4*, in the case of mortalities dynamics caused by injection into the adductor muscle, is related to the deletion of the *virB4* gene, thus demonstrating an important role for the T4SS in the virulence of *V. tapetis*.

## Discussion

Brown Ring Disease (BRD) primarily affects the Manila clam *R. philippinarum* and is due to an infection by *V. tapetis*, a bacterium that acts as an external microparasite to induce this disease (Paillard, 2004a). *V. tapetis* colonizes and degrades the periostracal lamina, in order to enter the extrapallial fluids (EPFs). During this process, a brown organic deposit is produced that results from the inhibition of shell biomineralization. This inhibition leads to accumulation of the organic part of the shell, the conchyoline that forms a brown ring inside the shell, giving its name to BRD (Paillard et al., 1994). Invasion of *V. tapetis* into EPFs also leads to several perturbations in the immune response capacities of the Manila clam, exemplified by a phenotype of rounded hemocytes (Choquet et al., 2003; Le Bris et al., 2015). Indeed, it has been demonstrated that *V. tapetis* induces a loss of adhesion properties of infected hemocytes and a standardized test have been developed in order to show and measure this phenotype *in vitro* (Choquet et al., 2003). Studies have also demonstrated that *V. tapetis* is able to modify processes such as phagocytosis, ROS production and resistance capacities of the hemocyte cells during infection (Paillard, 2004b; Allam and Ford, 2006; Jeffroy and Paillard, 2011). These modifications of hemocyte physiology are accompanied by actin cytoskeleton re-organization, as has been shown by analyses of deregulated genes in clams during infection or physiological assay (Paillard, 2004b; Brulle et al., 2012; Allam et al., 2014; Rahmani et al., 2019).

Nevertheless, almost nothing is known about the molecular mechanisms used by the pathogen in order to induce the phenotypes described above. Interestingly, a recent genomic comparison of *V. tapetis* strains highlighted the probable involvement of T4SS in BRD (Dias et al., 2018). Dias et al. compared the genomes of seventeen strains of *V. tapetis* isolated from either bivalves or fish. They highlighted that strains of *V. tapetis* pathogenic to clams are the only ones that carry a cluster of 11 genes encoding a VirB/D4 T4SS. It is therefore likely that the T4SS is involved in this ability of strains that are pathogenic to Manila clams, to induce BRD. T4SSs are found in nearly half of bacterial genomes (Guglielmini et al., 2011) and are implicated in the conjugation of DNA and/or the secretion of effectors into prokaryotic and/or eukaryotic cells. Its molecular structure is also very different depending on the infection strategy implemented by the species that carries this cluster, and on the bacterial species itself. The T4SS genes organization of *V. tapetis* CECT4600 is very close to the VirB/D4 system of *A. tumefaciens* (Dias et al., 2018).

Given that T4SSs are present in many pathogens and that a T4SS is present only in *V. tapetis* strains pathogenic to clams (Bidault et al., 2015; Dias et al., 2018), we decided to inactivate this system in *V. tapetis* CECT4600. To achieve this goal, we focused our effort on the deletion of the *virB4* gene of *V. tapetis* CECT4600. The VirB4 protein is one of the two ATPase of this T4SS and is involved in pilus biogenesis, as it provides the energy required for this process. The other ATPase, VirB11, functions with VirB4 to induce a structural change in the pilin and was thus suggested to modulate the VirB4 dislocase activity (Waksman, 2019).

Unfortunately, no efficient technique of gene deletion was available for *V. tapetis*, with only one study that was able to perform a gene deletion on *V. tapetis.* In that study Lakhal et al. (2008) succeeded more than 10 years ago to delete the *djlA* gene (849 bp), encoding a membrane-anchored DnaJ-Like protein that is involved in the rounding phenotype observed and measured by *in vitro* assay. *V. tapetis δdjlA* was not able to induce BRD in infected clams. However, the fact that this strain exhibits a significantly reduced growth rates makes its role in the virulence of *V. tapetis in vivo* undetermined (Lakhal et al., 2008). In this study, we succeeded in deleting the *virB4* gene, using a derivative of the pLP12 suicide plasmid (Luo et al., 2015). To achieve this, we developed a mutagenesis protocol specifically designed for *V. tapetis*, by identifying the appropriate mating temperature allowing recombination of the suicide plasmid into the *V. tapetis* genome. Despite this being a low frequency occurrence, performing the matings at 25°C was key to obtain one transconjugant in which the suicide plasmid has recombined within the genome of *V. tapetis* CECT4600. This temperature is sublethal for *V. tapetis* CECT4600, as this strain can survive but is not able to grow at this temperature. This stressful condition is proposed to alter potential restriction/modification systems (Rolfe and Holloway, 1969), making these stressed cells more prone to accept foreign DNA. An electroporation protocol has recently been developed to introduce foreign DNA in various marine bacteria, and successful transformation was only obtained if cells were grown at supraoptimal temperature (Delavat et al., 2018).

*V. tapetis* is able to grow and develop in clam hemocytes after phagocytosis (Paillard, 2004a; Balbi et al., 2018). This ability to grow in hemocytes may be due to the activity of the T4SS, as demonstrated with various pathogens. Indeed, *Legionella pneumophila*, that induces the Legionnaire’s disease in humans, carries a specialized Dot/Icm T4SS. This T4SS induces the formation of a *Legionella* containing vacuole (LCV) and escapes fusion with the lysosome (Grohmann et al., 2018). The *L. pneumophila* T4SS structure is close to the *Helicobacter pylori* Cag T4SS (Ghosal et al., 2017), and the former system allows the translocation of more than 300 effectors in eukaryotic cells, affecting pathways controlling membrane transport processes (Sherwood and Roy, 2016; Grohmann et al., 2018). The LCV is able to fuse with endoplasmic reticulum and to move along microtubules to deregulate factors for host signaling. Nevertheless, in this study we demonstrated that the amount of acidic organelles, which might be a proxy of lysosomal activity in this model (Rahmani et al., 2019), decreases after challenge of hemocytes by *V. tapetis* in the WT strain but also in the *virB4* mutant (**Figure 3**). Thus, although there is a probable link between intracellular survival and decrease of lysosomal activity, this process is not strictly dependent on the T4SS in the case of *V. tapetis* (**Figure 4**), which might reveal that a synergy of different mechanisms could be used by the pathogen to achieve this function.

The diversion of the actin cytoskeleton has already been shown in clam hemocytes following exposure to *V. tapetis* (Paillard, 2004b; Rahmani et al., 2019). Such a phenomenon has also been reported in other pathogenic species, forming a vacuole and encoding a T4SS. For example, *H. pylori*, that carries a *cag* pathogenicity island, and causes gastric ulcers and gastric adenocarcinoma in humans, secretes a protein, CagA, associated with the surface-exposed portion of the pilus (Backert et al., 2015). This effector is associated with the protein HtrA to open cell-to-cell junctions and to bind to the basolateral host receptor integrin. These proteins disorganize the apical junctions in stomach cells by a disorganization of the actin cytoskeleton (Selbach et al., 2003; Grohmann et al., 2018). *Bartonella henselae* carries a VirB/D4 type of T4SS which is involved in a reorganization of the cytoskeleton that leads to uptake of bacteria into endothelial cells (Grohmann et al., 2018). Finally, *Coxiella burnetii*, that causes Q-fever in humans, forms a Coxiella-containing-vacuole (CCV), using a Dot/Icm-like T4SS similar to that of *L. pneumophila*. It modifies host endocytic transport systems and replicates in CCVs interacting with the autophagosome (Kohler et al., 2016). Unlike *V. tapetis*, *C. burnetii* grows in a CCV that has acquired lysosomal characteristics such as acidic pH, acid hydrolases and cationic peptides (Voth and Heinzen, 2007). Interestingly*, in vitro* incubation of *R. philippinarum* hemocytes with *V. tapetis* heated cultures had revealed that the severe changes that affect hemocytes during exposure to *V. tapetis* (i.e. changes in structure and decrease in hemocyte phagocytic activity and viability) are related to a thermosensitive molecule (Allam and Ford, 2006). Thus, *V. tapetis* seems to possess thermosensitive cytotoxic factor(s) mediated by intact bacterial cells, which are also present as extracellular secretions (Allam and Ford, 2006) and might be secreted through the T4SS or even maybe a thermosensitive export system. In the dynamics of BRD, disorganization of hemocyte adhesion capacities is followed by an absence of phagolysosome fusion, thus allowing *V. tapetis* growth in the infected hemocytes in a vacuole formed after phagocytosis (Paillard, 2004b). Nevertheless, we demonstrated in this study that T4SS is not implicated in the cytotoxic effect of *V. tapetis* toward hemocytes, as *in vitro* assays showed that a *virB4* null mutant still induces a loss of hemocyte adhesion properties (**Figure 3**). Furthermore, since the VirB4 protein is involved in the pilus formation (Waksman, 2019), the predicted absence of a pilus in the *virB4* mutant shows that the T4SS pilus is probably not involved in this phenotype.

Finally, we could demonstrate that the deletion of the *virB4* gene in *V. tapetis* CECT4600, and therefore inactivation of its T4SS, significantly reduced BRD prevalence by 50% when compared to the WT strain (**Figure 5**). Furthermore, clam mortality dynamics and rates after injection of the adductor muscle significantly decreased in *V. tapetis* Δ*virB4*, as compared to the WT strain (**Figure 6**). We did not test whether the complemented strain is able to restore the prevalence of BRD phenotype induced by the WT (**Figure 5**), because we wanted to avoid the massive overuse (and sacrifice) of animals in our study (150 animals for an experiment reproducing BRD against 30 animals for an experiment reproducing septicemic mortalities, for each condition). However, we did construct a complemented mutant of *virB4*, which restored the mortality dynamics and partially this mortality rates (**Figure 6**). We hypothesize that this only partial complementation of mortality rate is due to the ectopic expression of the virB4 gene in our complemented strain.

Indeed, due to the lack of other genetic tools (like mini-transposons) available for this strain, we cloned the *virB4* gene in a replicative plasmid. Complementation using a replicative plasmid as the caveat that the number of plasmid copies cannot be controlled, influencing the regulation of the *virB4* gene expression and the number of VirB4 molecules. This, together with the production of *virB4* using the Plac promotor and RBS of the plasmid, may explain this only partial restoration of the virulence phenotype. All in all, we showed that the T4SS has a direct involvement in the virulence and BRD formation by *V. tapetis*. Interestingly, previous studies focusing on *V. tapetis* LP2, a strain that does not carry the T4SS cluster, might help us to understand the role of T4SS in the development of BRD. The LP2 strain has been isolated from the fish *Symphodus melops* (Jensen et al., 2003) and is not able to induce BRD in the classical way of infection (i.e. injection into the pallial cavity or balneation) (Choquet, 2004; Bidault et al., 2015; Dias et al., 2018). However, if this strain is injected directly into the extrapallial cavity of Manila clams, it can induce BRD at rates of 75%, as compared to strain CECT4600 injected into extrapallial cavity that had induced 100% of BRD is this particular study (Le Bris et al., 2015).

Considering the knowledge about other bacterial species encoding T4SS, it is likely that *V. tapetis* is able to secrete effector proteins during the dynamics of BRD. Nevertheless, because a *virB4* mutant is not able to reduce loss of adhesion properties in infected hemocytes, and because prevalence of BRD is reduced in the absence of the v*irB4* gene, we hypothesize that *V. tapetis* T4SS would probably be involved in the colonization of the host, thus suggesting that T4SS-pilus-mediated colonization is a key factor for the specificity of these pathogenic strains of *V. tapetis*, and in our study the strain CECT4600, toward the Manila clam. The BRD owes its name from the brown organic deposit observed during infection by *V. tapetis*. This reaction of the Manila clams to infection occurs in two steps: conchiolin deposit and shell process repair to enrobe bacteria (Paillard and Maes, 1995). It has also been shown that *V. tapetis* is able to disrupt the host biomineralization process to facilitate colonization (Trinkler et al., 2011). Indeed, *V. tapetis* colonizes the periostracal lamina and the epithelia of mantle fold (Paillard, 2004b). The mantle edge cells are involved in pathogen recognition and the “nacrezation” defense mechanism with the secretion of melanized matrices (Paillard et al., 1994; Allam et al., 2014; Allam and Raftos, 2015). Thus, we could hypothesize that the T4SS interacts with the shell biomineralization process by injecting effectors into the cytosol of mantle edge cells. Considering our study, the Δ*virB4* strain is able to colonize (at least partially) the periostracal blade and epithelium of the mantle edge, but it might induce a weaker immune response because this strain might be less recognized by the mantle cell receptors and therefore induces less brown deposit characterizing BRD.

Future analyses will focus on colonization of the periostracal lamina as observed by light and electronic microscopy but also on phenoloxydase production in mantle and extrapallial fluids, in order to test these hypotheses (Paillard, 2004a; Le Bris et al., 2015).

## Conclusions

In this study, we showed that the T4SS plays an important role in the dynamics of BRD but is not essential for BRD development. Considering our results and the knowledge on other pathogens carrying T4SSs, we hypothesize that this system might be involved in colonization steps or in the pathogen’s induction of the immune response in the development of BRD. Both of these hypotheses might be consistent with the reduced prevalence of BRD observed *in vivo*.

Gene deletion has been reported before, but in that study, only *in vitro* virulence assays were interpretable. Therefore, this study is the first gene deletion approach on *V. tapetis* that unveils an important molecular mechanism involved in the pathogenic activity of this bacterium in the BRD and not only on *in vitro* markers. Furthermore, it is the first study that confirms the involvement of T4SS in the development of BRD.

## Data Availability Statement

The raw data supporting the conclusions of this article will be made available by the authors, without undue reservation.

## Author Contributions

CP and VP acquired funds and coordinated the study. AR performed the experimental mutagenesis with the knowledge and help of FD. AR performed *in vitro* tests with the help of NL and CL for the flow cytometry analyses. AR performed *in vivo* infections technical design with the help of ED, NL, and CP. AR, FD, VP, and CP wrote the article (the original draft was written by AR). The article was carefully reviewed by other co-authors, who all approved the final version. All authors contributed to the article and approved the submitted version.

## Funding

This project received grants from the H2020 European project “VIVALDI” (grant agreement N°678589). This work was also supported by the University of Western Brittany (UBO), the “investment for the future” programs LabexMER (ANR-10-LABX-19) and ISblue (ANR-17-EURE-0015).

## Conflict of Interest

The authors declare that the research was conducted in the absence of any commercial or financial relationships that could be construed as a potential conflict of interest.

## Supplementary Material

Supplementary Figure 1 PCR with primers 170513-170514 that amplified virB4 gene. A fragment of 173 bp is expected if the gene virB4 is present, no amplification is expected if the gene virB4 is absent. T-: negative control of PCR with sterile water.

Supplementary Figure 2 Cytograms and Fluorescence Peaks corresponding to results of count of non-adherent hemocytes (laser FITC, **Figure 3**) and amount of acidic organelles (laser APC, **Figure 4**) in the conditions: control FSSW, V. tapetis CECT4600 and *V. tapetis ΔvirB4*. Flow repository ID: FR-FCM-Z3WT. **(A–C)**: Green fluorescence (FITC) in function of granularity (SSC) of the cells that belong to a region corresponding of total non-adherent hemocytes in the conditions. This region was defined to target hemocytes. Since our experiment only harvested non-adherent hemocytes, this region includes all the non-adherent hemocytes of the sample in the condition tested. **(A)** control FSSW, **(B)** *V. tapetis* CECT4600 and **(C)** *V. tapetis ΔvirB4*. **(E)** Distribution of the green fluorescence (FITC) of the total hemocytes count (same region as in figures **A–C**), in the conditions: control FSSW, *V. tapetis* CECT4600 and *V. tapetis ΔvirB4*. **(F)** Distribution of the red fluorescence (APC) of the total hemocytes count (same region as in figures **A–C**), in the conditions: control FSSW, *V. tapetis* CECT4600 and *V. tapetis ΔvirB4*.

Supplementary Figure 3 Picture of a Brown Ring Disease deposit on an infected Manila clam of the experiment described in **Figure 5**. **(A)** 1 valve pictured without a zoom. **(B, C)** Picture realized with a binocular loupe. The black arrows point the brown deposit that characterizes BRD at different zoom.

## Abbreviations

BRD, Brown Ring Disease; CFU, Colony Forming Unit; EPFs, Extrapallial Fluids; FSSW, Filter Sterilized Sea Water; LBS, Luria Broth Salted; PCR, Polymerase Chain Reaction; ROS, Reactive Oxygen Species; T4SS, Type IV Secretion System; WT, Wild Type.
