# Supplementary figures and images for: Implication of the Type IV Secretion System in the Pathogenicity of Vibrio tapetis, the Etiological Agent of Brown Ring Disease Affecting the Manila Clam Ruditapes philippinarum
Source: Front Cell Infect Microbiol. 2021 Apr 29;11:634427. doi: 10.3389/fcimb.2021.634427 (PMC8116749; doi:10.3389/fcimb.2021.634427)

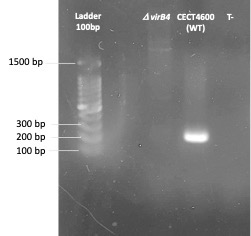

Supplement: Supplementary Figure 1 — PCR with primers 170513-170514 that amplified virB4 gene. A fragment of 173 bp is expected if the gene virB4 is present, no amplification is expected if the gene virB4 is absent. T-: negative control of PCR with sterile water. [file Image_1.jpeg]

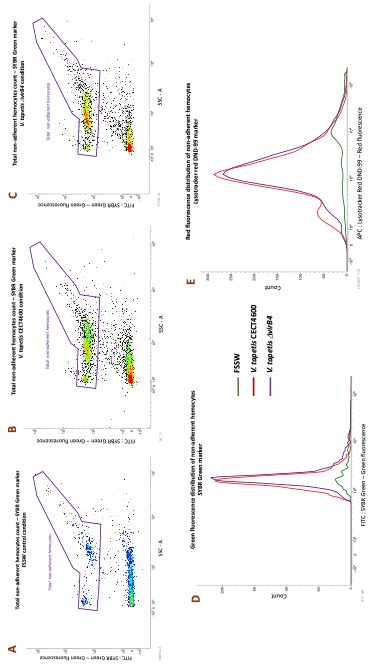

Supplement: Supplementary Figure 2 — Cytograms and Fluorescence Peaks corresponding to results of count of non-adherent hemocytes (laser FITC, Figure 3 ) and amount of acidic organelles (laser APC, Figure 4 ) in the conditions: control FSSW, V. tapetis CECT4600 and V. tapetis ΔvirB4. Flow repository ID: FR-FCM-Z3WT. (A–C): Green fluorescence (FITC) in function of granularity (SSC) of the cells that belong to a region corresponding of total non-adherent hemocytes in the conditions. This region was defined to target hemocytes. Since our experiment only harvested non-adherent hemocytes, this region includes all the non-adherent hemocytes of the sample in the condition tested. (A) control FSSW, (B) V. tapetis CECT4600 and (C) V. tapetis ΔvirB4. (E) Distribution of the green fluorescence (FITC) of the total hemocytes count (same region as in figures A–C), in the conditions: control FSSW, V. tapetis CECT4600 and V. tapetis ΔvirB4. (F) Distribution of the red fluorescence (APC) of the total hemocytes count (same region as in figures A–C), in the conditions: control FSSW, V. tapetis CECT4600 and V. tapetis ΔvirB4. [file Image_2.jpeg]

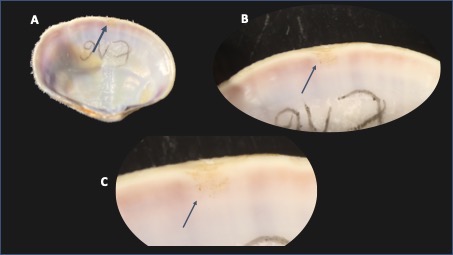

Supplement: Supplementary Figure 3 — Picture of a Brown Ring Disease deposit on an infected Manila clam of the experiment described in Figure 5 . (A) 1 valve pictured without a zoom. (B, C) Picture realized with a binocular loupe. The black arrows point the brown deposit that characterizes BRD at different zoom. [file Image_3.jpeg]
