# Supplementary material for: Implication of the Type IV Secretion System in the Pathogenicity of Vibrio tapetis, the Etiological Agent of Brown Ring Disease Affecting the Manila Clam Ruditapes philippinarum
Source: Front Cell Infect Microbiol. 2021 Apr 29;11:634427. doi: 10.3389/fcimb.2021.634427 (PMC8116749; doi:10.3389/fcimb.2021.634427)
Supplement: Supplementary file 4 [file Table_1.pdf]

Table S1 : Organization of T4SS virB/D4 gene cluster in five bacterial pathogens.

*For each strains, the label of the virB/D gene on MaGe : MicroScope plateform (Vallenet et al., 2009) was reported as well as the length of the gene. This table corresponds to the clusters represented in Fig. 1 of the manuscript. *hypothetical virB5 gene*

**virB/D4 genes and homologues Label and Length (bp) of the sequence on MaGe**	***Agrobacterium tumefaciens***		***Vibrio tapetis CECT4600***		***Bartonella henselae***		***Helicobacter pylori***		***Bordetella pertussis***	
virB1	ATAM_0148	720	-	-	-	-	-	-	-	-
virB2	ATAM_0149	366	VTAP_v1_a3553	321	BH13260	324	HPSP_04625	303	BP3788	309
virB3	ATAM_0150	327	VTAP_v1_a3552	372	BH13270	312	HPSP_04620	261	BP3789	315
virB4	ATAM_0151	2370	VTAP_v1_a3554	2526	BH13280	2355	HPSP_04615	2499	BP3790	2475
virB5	ATAM_0152	663	VTAP_V1_a3555*	792*	BH13290	447	-	-	-	-
virB6	ATAM_0153	888	VTAP_v1_a3557	948	BH13300	966	-	-	BP3791	1392
virB7	ATCDS0156888D	168	-	-	BH13310	411	-	-	BP3792	186
virB8	ATAM_0154	693	VTAP_v1_a3558	717	BH13320	669	HPSP_04600	987	BP3793	702
virB9	ATAM_0155	882	VTAP_v1_a3559	855	BH13330	864	HPSP_04595	1542	BP3794	822
virB10	ATCDS0159596D	1134	VTAP_v1_a3560	1254	BH133340	1191	HPSP_04590	1236	BP3795	1125
virB11	ATAM_0156	1032	VTAP_v1_a3565	747	BH13350	1071	HPSP_04565	942	BP3796	1020
virD4	ATAM_0170	1971	VTAP_v1_a3562	744	BH13380	1920	HPSP_04545	1728	-	-
References for T4SS organization	Stachel et Nester, 1986		Dias et al., 2018		Schröder et Dehio, 2005		Yuan et al., 2018		Wallden et al., 2010	
Name of the strain used in MaGe database	- plasmid AF242881.1		-chromosome A.1		Houston-1 - chromosome NC_005956.1		Shi470 - chromosome NC_010698.1		Tohama I - chromosome NC_002929.2	

Dias, G.M., Bidault, A., Le Chevalier, P., Choquet, G., Der Sarkissian, C., Orlando, L., Medigue, C., Barbe, V., Mangenot, S., Thompson, C.C., Thompson, F.L., Jacq, A., Pichereau, V., Paillard, C., 2018. *Vibrio tapetis* Displays an Original Type IV Secretion System in Strains Pathogenic for Bivalve Molluscs. Frontiers in microbiology 9, 227.

Schröder, G., Dehio, C., 2005. Virulence-associated type IV secretion systems of *Bartonella*. Trends in Microbiology 13, 336–342. https://doi.org/10.1016/j.tim.2005.05.008

Stachel, S.E., Nester, E.W., 1986. The genetic and transcriptional organization of the vir region of the A6 Ti plasmid of *Agrobacterium tumefaciens*. The EMBO Journal 5, 1445–1454. https://doi.org/10.1002/j.1460-2075.1986.tb04381.x

Wallden, K., Rivera-Calzada, A., Waksman, G., 2010. Microreview: Type IV secretion systems: versatility and diversity in function: Diversity and versatility of the type IV secretion system. Cellular Microbiology 12, 1203–1212. https://doi.org/10.1111/j.1462-5822.2010.01499.x

Yuan, X.-Y., Wang, Y., Wang, M.-Y., 2018. The type IV secretion system in *Helicobacter pylori*. Future Microbiology 13, 1041–1054. https://doi.org/10.2217/fmb-2018-0038
