# Supplementary material for: Implication of the Type IV Secretion System in the Pathogenicity of Vibrio tapetis, the Etiological Agent of Brown Ring Disease Affecting the Manila Clam Ruditapes philippinarum
Source: Front Cell Infect Microbiol. 2021 Apr 29;11:634427. doi: 10.3389/fcimb.2021.634427 (PMC8116749; doi:10.3389/fcimb.2021.634427)
Supplement: Supplementary file 5 [file Table_2.docx]

Table S2: Primers used in this study, Eurofins Genomics ©

| **Primer Name** | **Sequence (5' -> 3')** | **Description** |
| --- | --- | --- |
| 170513 | TTAAAAGTGGCGGAGGAATG | Forward primer annealing within the *virB4* gene |
| 170514 | AAGCTCTGCATCGGTTAGGA | Reverse primer annealing within the *virB4* gene |
| 170607 | TTTTTGAATTCCACGTTCAAAACTATCCAAAG (*Eco*RI) | Forward primer to amplify the UP region of *virB4* gene |
| 170608 | GGGCTTGTGCATAATTAGAAC | Reverse primer to amplify the UP region of *virB4* gene |
| 170609 | *GTTCTAATTATGCACAAGCCC*CGGAACTAAGGAAAGAGCTG (reverse complement of primer 170608 in italic) | Forward primer to amplify the DOWN region of *virB4* gene |
| 170712 | TTTTTTGCTAGCGGTTTCATTAACGTCAATCTC (*Nhe*I) | Reverse primer to amplify the DOWN region of *virB4* gene |
| 170611 | GGTTAGAACTTTTCGAGGAAC | Forward primer annealing 33 bp upstream of the UP region of *virB4* gene |
| 170612 | CCAAATCGTGTTTGACGTTC | Reverse primer annealing 83 bp downstream of the DOWN region of *virB4* gene |
| 170515 | GTTTTCCCAGTCACGAC | Forward primer annealing 111 bp before pGEM-T insertion site |
| 170516 | CAGGAAACAGCTATGACC | Reverse primer annealing 126 bp after pGEM-T insertion site |
| 180915 | TTTTTTCTAGAAAGAAGGAGATATACATATGCACAAGCCCCTCAATAG (*Xba*I) | Forward primer amplifying *virB4* gene |
| 180916 | TTTTTTGGATCCTCACAGCTCTTTCCTTAGTTC (BamH1) | Reverse primer amplifying *virB4* gene |
| 170901 | GCTCGAGGGAATATAAGTCG | Forward primer annealing 59 bp before UD fragment in pFD055 |
| 170902 | CGGCTGACATGGGAATTGC | Reverse primer annealing 52 bp after UD fragment in pFD055 |
| 180401 | ACGTACTAGTGATATCCTGCAGCTCGAGGGTTCGCGTTGGCCGATTC (*Spe*I) | Forward primer annealing in Plac promotor in pFD086 |

Restriction sites are underlined
