# Supplementary material for: Implication of the Type IV Secretion System in the Pathogenicity of Vibrio tapetis, the Etiological Agent of Brown Ring Disease Affecting the Manila Clam Ruditapes philippinarum
Source: Front Cell Infect Microbiol. 2021 Apr 29;11:634427. doi: 10.3389/fcimb.2021.634427 (PMC8116749; doi:10.3389/fcimb.2021.634427)
Supplement: Supplementary file 6 [file Table_3.docx]

Table S3: Growth parameters of V. tapetis CECT4600 and V. tapetis ΔvirB4 in LBS and Zobell media, with shaking at 18°C in 100-well microplates.

|  |  | LBS | | | |  | | Zobell | | |
| --- | --- | --- | --- | --- | --- | --- | --- | --- | --- | --- |
| **Strains** | **T°** | Doubling Time (h) | Maximum OD _492_ | Lag Phase (h) |  | |  | Doubling Time (h) | Maximum OD _492_ | Lag phase (h) |
| *V. tapetis* CECT4600 | 18°C | 4.42 ± 0.04 | 1.74 ± 0.01 | 3.33 ± 0.00 |  | |  | 2.22 ± 0.19 | 1.29 ± 0.01 | 2.53 ± 0.18 |
| *V. tapetis* Δ*virB4* | 18°C | 6.00 ± 0.07 | 1.59 ± 0.01 | 3.40 ± 0.15 |  | |  | 2.55 ± 0.10 | 1.13 ± 0.01 | 2.25 ± 0.32 |
